# Injury to the Lower Bowel by the Accidental Introduction of the Handle of a Hoe into the Rectum

**Published:** 1871

**Authors:** R. W. Hargadine

**Affiliations:** Felton, Delaware


*Injury to the Lower Bowel by the Accidental Introduction of the Handle of a Hoe into the Rectum.* By R. W. Hargadine, M.D., Felton, Delaware, July 5th, 1871.

---

Ordinarily, the introduction of foreign bodies into the rectum, whether by accident or design, is not of sufficiently rare occurrence to deserve special notice, almost every surgeon of experience having met with examples of this kind in his practice. But the case, which I record below, reminds one of that celebrated case related by Marcletti, in the transactions of the Royal Academy of Surgery, of Paris, of the introduction of the large end of a pig's tail into the rectum of a courtesan. This unique weapon, remaining there for several days very nearly destroyed life.

Willie W., age 11 years, while jumping from the fence, June 15th, accidently landed on the end of a hoe-handle which he had planted in the ground, and over which, in company with a girl of about the same age, he had been performing a series of original gymnastic evolutions. The extremity of the handle passed through the seat of his breeches and into the rectum. He fell to the ground sweeping the implement down with him. The girl freed him from his unpleasant situation. He passed considerable blood from the bowel, but was able to walk to the house, a distance of several hundred yards. As he lived several miles in the country, I could not see him till about three hours after the accident. On my arrival I examined, first, the implement, and found it rough, somewhat splintery, and about one and a quarter inches in diameter, covered with blood and fecal matter to the distance of about eight inches from the extremity, showing that it had passed that distance into the bowel.

On examination I found the sphincter muscle ruptured slightly, anteriorly and posteriorly, with the rectum lying open, having apparently lost its contractile power, so much so that the eye could penetrate a distance of two or three inches into the bowel. So far as could be seen by the eye, the membrane appeared ragged and congested ; on passing my finger upward I found the mucous coat apparently roughened and in shreds. A *cul de sac* seemed to have been formed by the end of the handle as it passed upward and to the right side of the body, pushing the wall of the rectum before it. I could pass my finger up this sac toward the right ilium, without, however, reaching its termination. The natural continuation of the bowel could be traced to the other side, but evidently contracted. The foreign body appeared to have passed up the natural tract of the bowel for a distance of about four inches, and then formed a tract of its own, terminating in the neighborhood of the right groin. It was feared there might be perforation of the bowel, but subsequent events could alone determine the extent of the injury.

As to the general condition, the extremities were cold, the pulse frequent and feeble, and the patient complained of intense pain, which he referred to the super-pubic region and loins. Diffusible stimulants with morphia were at first used to promote reaction and relieve pain. Reaction took place in a few hours. One third of a grain of opium was then given every two hours to relieve the pain and "put the bowels in splints." Warm emollient applications were ordered to be used over the abdomen and cold wet cloths to the anus.

*June 16th.*—Patient still complains of pain in the right groin and lower abdominal region. Abdomen distended with gas, and tender, especially on the right side. Pulse 120, tongue thickly furred, no tendency to evacuate the bowels; urine passed without difficulty. The opium was still continued, with one-quarter grain of calomel in each dose. Injections of cold water were used to wash out the lower bowel and to prevent the inflammatory action from running too high.

*June 17th.*—Condition about the same as yesterday. Abdomen less tympanitic. A rounded swelling was observed in the right iliac region, exceedingly tender to the touch, and on movement of the body; skin hot and dry; tongue still thickly furred; vomited several times a greenish, billious-looking matter. A sero-purulent discharge from the rectum has set in, exceedingly fetid and profuse. Warm water injections were used with a weak solution of carbolic acid. These injections were intended only for the rectum and were not forced into the upper bowel. Treatment still continued; mild liquid food in small quantities allowed.

*June 18th.*—The patient in about the same condition as yesterday. Tongue somewhat dryish. The swelling in the right side still continues. In addition to opium, camphor was given. Had a few hours refreshing sleep last night.

*June 19th.*—To-day a natural evacuation from the bowels took place without much pain, but there was no control over the sphincter. Gave ol. ricini f℥iii, with ol. terebinth gtt. xv., pro ducing three evacuations, very fetid, and containing shreds of decomposing membrane.

*June 21st.*—But little tympanitis to-day; swelling and tenderness in right iliac region still continues; bowels relaxed; stools watery; tongue cleaning off and pulse abating.

*June 23d.*—Patient doing very well to-day. The pulse about ninety to the minute; skin moist and cool; tongue looking better. has less thirst and some appetite. The bowels are still relaxed and there is much tenderness in the right groin. In addition to opium, one grain of acetate of lead every four hours was given.

*June 30th.*—Sat up some to-day. The sphincter is contracting and the patient has pretty good control over it; the ruptured edges have united; the swelling in the right side is diminishing somewhat; it was ordered to be painted once daily with tr. iodine.

*July 4th.*—The patient has been up most of the time since I saw him last, and is doing very well. The iodine is still used externally, and a small dose of opium is given occasionally to keep the bowels in check. I omitted to mention that an hour or two before the receipt of the injury, the boy had eaten very freely of cherries, swallowing the stones; these he vomited before my arrival. There was marked disturbance of the digestive organs in this case, and some of the symptoms may perhaps be referred to the presence of indigestible matter in the alimentary canal; and had the nature of the case not been so evident, the swelling and tenderness in the right iliac region might have been mistaken for typhlitis, produced by the presence of fruit-stones in the vermiform appendix, or impacted matters in the neighborhood of the ilio-cœcal valve.

